# Role of Hyaluronan in Inflammatory Effects on Human Articular Chondrocytes

**DOI:** 10.1007/s10753-019-01043-9

**Published:** 2019-06-27

**Authors:** Mary K. Cowman, Claire Shortt, Shivani Arora, Yuhong Fu, Jemma Villavieja, Jai Rathore, Xiayun Huang, Tatini Rakshit, Gyu Ik Jung, Thorsten Kirsch

**Affiliations:** 1 Department of Biomedical Engineering, New York University Tandon School of Engineering, 433 First Avenue, room 910, New York, NY 10010 USA; 2 Musculoskeletal Research Center, Department of Orthopaedic Surgery, New York University School of Medicine, New York, NY USA; 3 Department of Chemical and Biomolecular Engineering, New York University Tandon School of Engineering, New York, NY USA

**Keywords:** hyaluronan, hyaluronidase, articular cartilage, chondrocytes, inflammation

## Abstract

Hyaluronan (HA) fragments have been proposed to elicit defensive or pro-inflammatory responses in many cell types. For articular chondrocytes in an inflammatory environment, studies have failed to reach consensus on the endogenous production or effects of added HA fragments. The present study was undertaken to resolve this discrepancy. Cultured primary human articular chondrocytes were exposed to the inflammatory cytokine IL-1β, and then tested for changes in HA content/size in conditioned medium, and for the expression of genes important in HA binding/signaling or metabolism, and in other catabolic/anabolic responses. Changes in gene expression caused by enzymatic degradation of endogenous HA, or addition of exogenous HA fragments, were examined. IL-1β increased the mRNA levels for HA synthases HAS2/HAS3 and for the HA-binding proteins CD44 and TSG-6. mRNA levels for TLR4 and RHAMM were very low and were little affected by IL-1β. mRNA levels for catabolic markers were increased, while type II collagen (α1(II)) and aggrecan were decreased. HA concentration in the conditioned medium was increased, but the HA was not degraded. Treatment with recombinant hyaluronidase or addition of low endotoxin HA fragments did not elicit pro-inflammatory responses. Our findings showed that HA fragments were not produced by IL-1β-stimulated human articular chondrocytes in the absence of other sources of reactive oxygen or nitrogen species, and that exogenous HA fragments from oligosaccharides up to about 40 kDa in molecular mass were not pro-inflammatory agents for human articular chondrocytes, probably due to low expression of TLR4 and RHAMM in these cells.

**Electronic supplementary material:**

The online version of this article (10.1007/s10753-019-01043-9) contains supplementary material, which is available to authorized users.

## INTRODUCTION

The effects of changes in the molecular mass (M) of hyaluronan (HA) in the pericellular environment during inflammatory processes are still incompletely understood. High M HA is considered a physiological protector of cells. It acts as a scaffold to assemble a matrix of proteoglycans, and it binds and clusters its cell surface receptors [1–3]. *Via* the receptors CD44 and/or ICAM-1, high M HA reduces the catabolic effects of interleukin-1β (IL-1β), tumor necrosis factor-alpha (TNF-α), and lipopolysaccharide (LPS) [4–6]. It also shields the cell from reactive oxygen and nitrogen species (ROS/RNS) generated under inflammatory conditions, by serving as a first target [7]. The products of such ROS/RNS action are fragments of the normally mega-Dalton HA molecules. HA fragments may also be produced by hyaluronidases shed into the inflammatory microenvironment [8, 9]. The relative rates of synthesis and degradation determine the content and size of HA outside the cell [10]. For many cell types, exogenous low M HA fragments have been shown to stimulate either defensive or pro-inflammatory cellular responses [7, 10–17]. The low M HA signaling involves toll-like receptor 4 (TLR4), toll-like receptor 2 (TLR2), CD44, receptor for hyaluronan-mediated mobility (RHAMM), or a number of other proteins whose complex binding interactions are altered by the de-clustering of CD44. The biological effects of low M HA can be cell type and context specific, because the levels of the relevant receptor or signal pathway proteins can vary. HA fragments failed to cause pro-inflammatory or other signaling effects in certain cell types [18, 19], and the medical use of a recombinant hyaluronidase enzyme does not cause an inflammatory response [20]. For articular chondrocytes in an inflammatory environment, published studies have failed to reach consensus on the endogenous production [21, 22] or effects of added low M HA fragments [3, 19, 23–28]. The present study was undertaken to help resolve this discrepancy.

We hypothesized that quantification and control of the size and concentration of HA fragments, as well as characterization of the expression levels of HA binding proteins, receptors, and synthetic/degradative enzymes would aid in clarifying the potential signaling role of HA fragments in articular chondrocytes. Cultures of primary human articular chondrocytes were exposed to the inflammatory cytokine IL-1β, and then tested for changes in HA content/size in conditioned medium, and for the expression of genes important in HA binding or metabolism, and in other catabolic/anabolic responses. In addition, changes in gene expression caused by enzymatic degradation of endogenous HA in chondrocyte cultures, or addition of exogenous low M HA fragments, were examined. The results show that HA fragments are not pro-inflammatory in cultured human articular chondrocytes that express low levels of the TLR4 and RHAMM receptors.

## MATERIALS AND METHODS

### Reagents

Polydisperse HA samples with weight-average M of 112 kDa and 22 kDa and having low endotoxin levels (≤ 0.004 EU/mg) were obtained from Lifecore Biomedical LLC (Chaska, MN). Low endotoxin (< 0.005 EU/mg) even-numbered HA oligosaccharides (4-mer, 6-mer, 8-mer) with N-acetyl-D-glucosamine at the reducing end were obtained from Contipro (Czech Republic). HA size standards for electrophoresis (Select-HA™ LoLadder and HiLadder) were obtained from Hyalose LLC (Austin, TX). Recombinant bovine PH-20 (rbPH-20, also called SPAM-1), Leu36-Thr497 with C-terminal 6-His tag (expressed in CHO cells, < 0.1 EU/μg) was from R&D Systems (Minneapolis, MN). Bovine testicular hyaluronidase (BTH, EC 3.2.1.35) was from Sigma-Aldrich (St. Louis, MO). Proteinase K was purchased from Roche Applied Science (Germany). Phosphate-buffered saline (PBS, 0.01 M phosphate, 0.138 M NaCl, 0.0027 M KCl, pH 7.4; catalog no. P3813) and endotoxin-free water were obtained from Sigma-Aldrich. Ethanol, 200 Proof, ACS Certified, was from Sigma-Aldrich and was filtered using a Whatman GD/X Sterile 0.2 μm CA filter (Thermo Fisher Scientific). All other chemical reagents were purchased from Sigma-Aldrich. Deionized water, 0.2 μm filtered, was from a MilliQ water purification system.

### Cell Culture

Human articular chondrocytes were isolated from articular cartilage samples obtained from patients (donor age range 48–67) undergoing total knee replacement surgery at NYU Langone Orthopedic Hospital. Knee cartilage was harvested from regions with no macroscopically evident degeneration. The possibility that cells from damaged tissue might have different expression profiles was not examined. The collection of tissue from patients undergoing knee replacement surgery was approved by the Institutional Regulatory Board at NYU School of Medicine. We combined the cartilage from two patients for each experiment. The potential effect of donor age on results was not examined. Human chondrocytes were isolated from these cartilage samples as described by us [29]. Cells were plated at density of 1.8 × 10^5^ cells/cm^2^ and grown in monolayer cultures in Dulbecco’s modified Eagle’s medium (DMEM; Life Technologies, Gaithersburg, MD) containing 25 mM glucose, with added 10% fetal calf serum (FCS; HyClone, Logan, Utah), 2 mM L-glutamine (Invitrogen, Carlsbad, CA), and 50 U/mL of penicillin and streptomycin (Invitrogen) (complete medium). These conditions have been shown to maintain their chondrocytic phenotype [30]. (The use of ascorbic acid was avoided because it is known to cause HA degradation, by reducing Fe^3+^ to Fe^2+^, which is active in production of hydroxyl radicals in the presence of hydrogen peroxide [7, 31]. Further, hydrogen peroxide formation is strongly increased by addition of ascorbic acid to DMEM buffer [32].) After cells have reached confluency, they were serum-starved for 24 h and then cultured in the presence of known (IL-1β, 10 ng/mL) or potential (HA fragments, 40 μg/mL) inflammatory stimuli in PBS containing 0.1% bovine serum albumin (BSA) for various time points. IL-1β at a concentration of 10 ng/mL was used because, as shown previously, this concentration was very effective in stimulating catabolic events and inhibiting anabolic events in human articular chondrocytes [33]. To test the effect of hyaluronidase digestion, serum-starved cells were cultured in the presence of rbPH-20 or BTH at various concentrations in PBS/0.1% BSA for 24 h.

### Reverse Transcription–PCR and Real-Time PCR Analysis

Total RNA was isolated from chondrocyte cultures using an RNeasy Mini kit (Qiagen, Valencia, CA). Levels of messenger RNA (mRNA) for aggrecan, bikunin, CD44, IL-6, HAS2, HAS3, HC1, HC2, HC3, Hyal2, iNOS, MMP-13, RHAMM, TLR2, TLR4, TSG-6, and type II collagen were quantified by real-time polymerase chain reaction (PCR) as previously described [34, 35]. Briefly, 1 μg of total RNA was reverse transcribed using the High Capacity cDNA synthesis kit (Applied Biosystems, Foster City, CA). A 1:100 dilution of the resulting cDNA was used as the template to quantify the relative content of mRNA by real-time PCR (ABI Stepone Plus; Applied Biosystems), using the appropriate primers and RT^2^ SYBR Green ROX FAST Mastermix (Qiagen). PCRs were performed at 95 °C for 10 min, followed by 40 cycles of 95 °C for 10 s and 60 °C for 30 s, and 1 cycle of 95 °C for 15 s and 60 °C for 1 min. The 18S RNA was amplified at the same time and used as an internal control. The cycle threshold values for 18S RNA and the samples were measured and calculated. Transcript levels were calculated according to the equation *x* = 2^−ΔCq^, where ΔCq = Cq_exp_ − Cq_18S_, or relative transcript levels were calculated as *x* = 2^−ΔΔCt^, in which ΔΔCt = ΔE − ΔC, ΔE = Ct_exp_ − Ct_18S_, and ΔC = Ct_ctl_ − Ct_18S._

### HA Purification and Analysis

Protein digestion is necessary for accurate analysis of the concentration and size of HA in conditioned medium from chondrocyte cultures. Briefly, 1 mL of conditioned medium was dialyzed (ThermoFisher Scientific mini dialysis unit, 2 mL volume, 3.5 kDa cutoff, catalog number 88403, or mini dialysis unit, 0.5 mL volume, 3.5 kDa cutoff, catalog number 88400) against 0.1 M NaCl overnight at room temperature. The volume after dialysis was determined by weight. Protein was digested using Proteinase K. The stock solution as supplied was 19 mg/mL in 10 mM Tris, pH 7.5, stabilized with calcium acetate, and 25 μL of the stock solution was used for each dialyzed sample (*ca.* 1 mL). Samples were incubated at 60 °C overnight. Proteinase was denatured by placing the sample in a boiling water bath for 15–20 min. For ELISA-like assay, samples were dialyzed against deionized water. Each sample was spiked with a pure HA standard of known concentration (100 ng/mL), to bring all samples into the optimum concentration range of the assay, and was accounted for in the result calculation. HA concentration was determined by a competitive ELISA-like assay (#K-1200, Echelon Biosciences, Salt Lake City, UT). ELISA assay plates were read on a Molecular Devices (Sunnyvale, CA, USA) SpectraMax® i3 Multi-Mode Microplate Detection Platform and analyzed with its SoftMax Pro software.

For analysis of the HA size distribution, purified samples (without spiked HA) were analyzed by agarose and polyacrylamide gel electrophoresis. Agarose gels of 0.5% in TBE buffer were run as described to analyze high M HA [36]. Polyacrylamide gels (4–20% polyacrylamide gradient containing constant 2.6% bisacrylamide, in Tris-borate-EDTA buffer, 1.0 mm × 10 well, ThemoFisher scientific catalog number EC622500X) were used to analyze HA below about 300 kDa [36]. As previously reported for HA isolated from human milk, non-GAG contaminants can be incompletely removed by the HA isolation procedure, and are observed in the agarose gels to bind dye but diffuse across gel lanes during staining and destaining [37].

### EV Isolation and Analysis

Samples of conditioned medium (300 μL) were diluted with 700 μL phosphate-buffered saline (PBS), and centrifuged at 2000 x g for 10 min. Pellets were discarded, and the sizes and numbers of extracellular vesicle (EV) present were assayed by nanoparticle tracking analysis (NTA), with a Malvern Nanosight LM10 Nanoparticle Tracking Analyzer.

### Preparation of Low Endotoxin HA Fragments

HA fragments of differing average molecular mass and low polydispersity were prepared by size fractionation of a polydisperse mixture of pure low endotoxin HA, using a step gradient anion exchange separation procedure essentially as previously described [37] but scaled up for a larger HA quantity, and performed under conditions to avoid endotoxin contamination. The columns (Pierce strong anion exchange spin columns, maxi size, #90011, from ThermoFisher Scientific, Waltham, MA) were pre-washed sequentially with 5 mL each of 70% ethanol, 0.10 M NaCl, 0.80 M NaCl (three times), and 0.10 M NaCl (five times). The HA sample to be fractionated was prepared by mixing 2.0 mL of a 0.5 mg/mL solution of 122 kDa HA in 0.10 M NaCl with 2.0 mL of a 0.5 mg/mL solution of 22 kDa HA in 0.10 M NaCl, and 6.0 mL of 0.10 M NaCl. The 10 mL polydisperse HA solution, containing 2 mg HA in 0.10 M NaCl, was loaded on the column and centrifuged. The column was washed with 5 mL of 0.10 M NaCl (once), then 5 mL of 0.2 M NaCl (three times). HA fragments were then eluted stepwise with sterile NaCl solutions at concentrations of 0.30, 0.33, 0.36, 0.40, 0.416, 0.44, and 0.80 M NaCl. At each step, the column was eluted twice with 2 mL of salt solution. The 4 mL eluates for each salt concentration were mixed at room temperature with 16 mL filtered absolute ethanol, and then divided into two sterile ultracentrifuge tubes, capped and stored at − 20 °C overnight to precipitate the HA. After centrifugation at 17,000×*g* for 50 min at 4 °C, the supernatant was removed with a pipette. The HA pellet was mixed with *ca.* 8 mL of absolute ethanol, stored at − 20 °C overnight, then centrifuged again at 17,000×*g* for 50 min at 4 °C. The supernatant was removed and the HA pellet was air-dried for at least 3 h in the biosafety hood. HA was redissolved in endotoxin-free water, with a target concentration of approximately 1 mg/mL. Actual HA concentrations were determined using a competitive ELISA-like assay (Echelon Biosciences). Endotoxin was assayed using the LAL (Limulus amebocyte lysate) Chromogenic Endotoxin Quantitation kit (#88282) from Thermo Scientific. The molecular mass distribution of each HA fraction was determined by 4–20% gradient polyacrylamide gel electrophoresis (PAGE) as previously described [36].

### Statistical Analysis

Student’s *t* tests were performed to evaluate differences between 2 groups; analysis of variance was performed to evaluate differences among > 3 groups. Tukey’s multiple comparison test was applied as a *post hoc* test. *P* values less than 0.05 were considered significant.

## RESULTS

### IL-1β Altered Chondrocyte Expression of Genes for Hyaluronan Synthases and HA-Binding Proteins

We first confirmed that treatment of our human articular chondrocyte cultures with IL-1β, one of the main catabolic and inflammatory cytokines in OA, resulted in the expected marked increase in mRNA levels of catabolic and inflammatory markers, including Cox-2, IL-6, iNOS, and MMP-13, and a decrease in expression of anabolic cartilage markers, including aggrecan and type II collagen (α1(II)) (see also Fig. 7) [4, 22, 28, 38, 39].

**Fig. 1:**
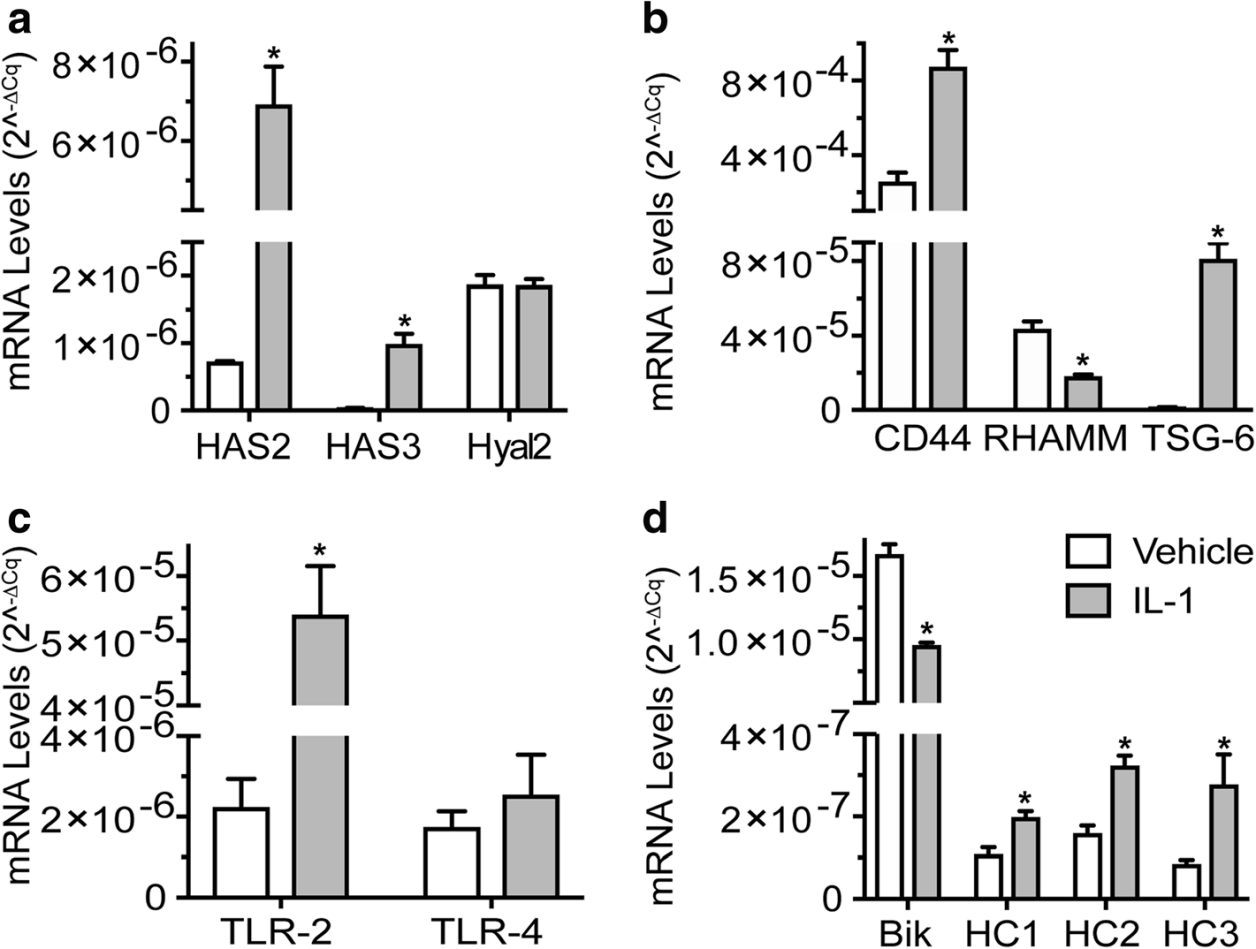
Effect of IL-1β treatment on mRNA levels for HA synthase enzymes (HAS2, HAS3), HA-degrading enzyme (Hyal2) (**a**), HA-binding proteins (CD44, RHAMM, TSG-6) (**b**), putative HA-binding proteins (TLR2, TLR4) (**c**), and HA-modifying proteins (Bikunin (Bik), HC1, HC2, HC3) (**d**) in cultured human articular chondrocytes. Human articular chondrocytes cultured in serum-free medium were treated with IL-1β at 10 ng/mL in PBS/0.1%BSA (IL-1), or PBS/0.1% BSA alone (vehicle) for 24 h. mRNA levels were determined by real-time PCR using SYBR Green and normalized to 18S RNA. Data were obtained from triplicated PCR reactions using RNA from three different cultures. Values are the mean ± SD (*p < 0.01).

IL-1β treatment also altered expression of genes for proteins important in HA metabolism, binding, and signaling. In agreement with previous reports [40, 41], we observed the mRNA levels for the hyaluronan synthases HAS2 and HAS3 to be increased by IL-1β. The mRNA level for the degradative hyaluronidase enzyme Hyal2 was unchanged in our studies, although an increase had been reported elsewhere [42]. mRNA for Hyal1 was present at only very low level in these chondrocytes, and mRNA for two other HA-degradative enzymes CEMIP (also known as KIAA1199, HYBID) and PH-20 were tested but not detected (Fig. 1a).

Among HA-binding proteins, mRNA levels were decreased for aggrecan in IL-1β-treated chondrocytes, as noted above, but strongly increased for TSG-6. Expression of the gene for CD44 was also increased, as noted in other studies [22, 40, 43], while RHAMM expression was decreased (Fig. 1b).

For the putative HA-binding TLRs, mRNA for TLR2 was increased as previously reported [44–46], while mRNA for TLR4 was present at only very low levels in the human articular chondrocytes studied here, and the expression levels were not increased by IL-1β treatment (Fig. 1c). The expression of TLR4 in human chondrocytes has previously been reported to be absent or variable [44, 45] and may be patient specific. A recent report suggests that glucose levels in the medium used for horse chondrocyte pellet cultures can significantly affect TLR4 expression, with high glucose (25 mM, as used in the present study) being associated with low levels of mRNA for TLR4 [47].

The increase in gene expression for TSG-6 suggested the importance of examining expression levels for genes related to inter-α-inhibitor (IαI). IαI is a complex proteoglycan structure composed of the core protein bikunin and a single covalently attached chondroitin sulfate chain, which is itself covalently modified by attachment of one to three proteins called heavy chains (HCs) 1, 2, and 3. TSG-6 is able to catalyze the transfer of HC domains from IαI to HA, where HC-HC interactions act to crosslink HA. Bikunin was highly expressed by human articular chondrocytes, but expression was reduced about 40% for chondrocytes cultured with IL-1β. Heavy chains HC1, HC2, and HC3 were all expressed at low levels in vehicle-treated chondrocytes, but increased in the presence of IL-1β (Fig. 1d). The mismatch of bikunin and HC expression patterns suggests the possible presence of a non-bikunin analog of IαI, as has been proposed [48], and the potential for covalent modification of HA by HC, especially under IL-1β treatment.

### HA Concentration in Conditioned Medium Was Increased by IL-1β Treatment

**Fig. 2:**
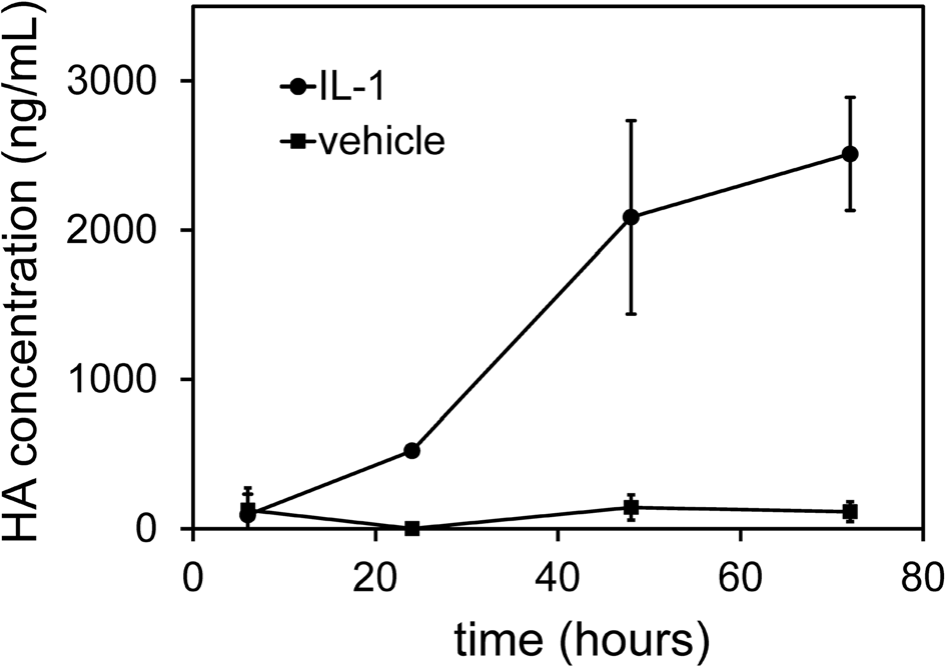
HA concentration in conditioned medium from cultured human articular chondrocytes was increased by IL-1β treatment. Human articular chondrocytes cultured in serum-free medium were treated with IL-1β at 10 ng/mL in PBS/0.1%BSA (IL-1), or PBS/0.1% BSA alone (vehicle) for increasing periods of time. HA was isolated and assayed using a specific ELISA-like assay, using three samples each of conditioned medium from IL-1 or vehicle treated chondrocytes, and are expressed as mean ± SD.

As expected from the IL-1β-induced increase in HAS2/HAS3 expression, but constant Hyal2 expression, the concentration of HA in chondrocyte conditioned medium was increased relative to vehicle-treated cells, and increased with duration (6–72 h) of IL-1β (10 ng/mL) treatment (Fig. 2).

### The Number of EVs Released into Conditioned Medium by Cultured Chondrocytes Was Increased by IL-1β Treatment

Treatment of cultured human articular chondrocytes with IL-1β caused an approximately twofold increased release of EVs into the conditioned medium. After removal of cell debris by centrifugation, EVs in the conditioned medium were counted by nanoparticle tracking analysis (NTA) (Supplementary Fig. 1). These EVs may be either exosomes, released by fusion of cytoplasmic multivesicular bodies with the plasma membrane, or microvesicles, formed directly from plasma membrane protrusions. HA is not known to affect the production or release of exosomes, but increased HA synthesis at the cell surface can drive the formation of microvesicles [49], suggesting that the increased number of EVs following IL-1β treatment may reflect in part the increased HA production.

### HA in Conditioned Medium from IL-1β-Treated Chondrocytes Retained a High Molecular Mass (M)

**Fig. 3:**
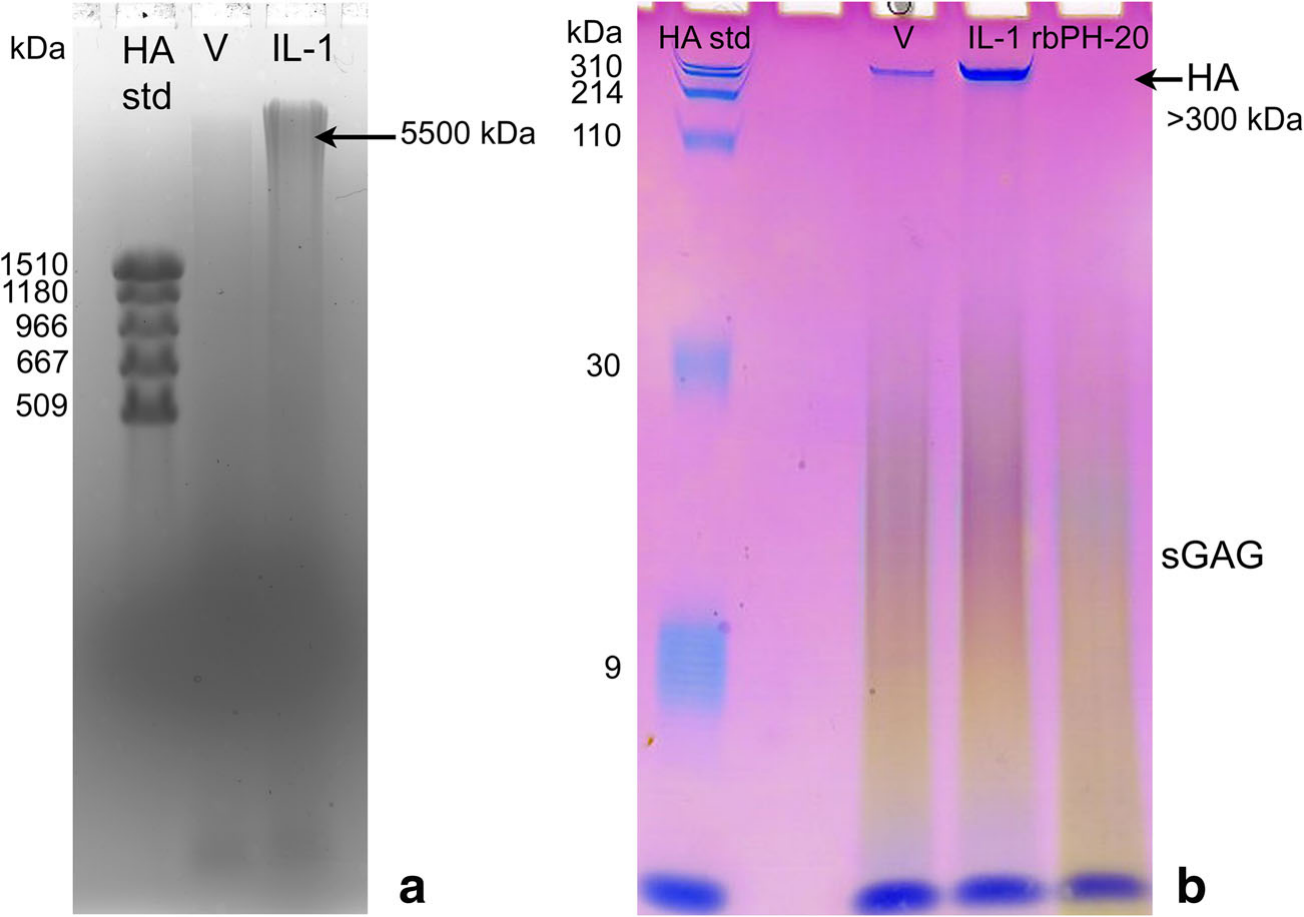
HA from conditioned medium of IL-1-treated human articular chondrocytes was very high in molecular mass but was completely absent in medium from cultures treated with recombinant bovine PH-20 (rbPH-20) hyaluronidase. Human articular chondrocytes cultured in serum-free medium were treated with IL-1β at 10 ng/mL in PBS/0.1%BSA (IL-1), or PBS/0.1% BSA alone (vehicle, V), or 5 μg/mL rbPH-20 in PBS/0.1% BSA for 24 h. high M HA was analyzed by electrophoresis on 0.5% agarose gel (**a**). Low M HA was analyzed by electrophoresis on 4–20% gradient polyacrylamide gel (**b**). A sharp blue band due to HA larger than about 300 kDa is seen in the PAGE gel for conditioned medium from IL-1-treated and vehicle treated cells but is absent from cells treated with rbPH-20 hyaluronidase. Gels were stained with Stains-All dye, differentiating HA (blue) from sulfated glycosaminoglycans (sGAGs) (stained purple to yellow). The blue band at the gel bottom is due to added bromophenol blue tracking dye.

The M distribution of HA purified from conditioned medium from human articular chondrocytes was determined by electrophoresis in 0.5% agarose gel (best for high M HA) and in 4–20% polyacrylamide gradient gel (PAGE) (best for low M HA) (Fig. 3). On agarose gel electrophoresis, HA from conditioned medium of IL-1β-treated chondrocytes was found to have very high average M (peaking at *ca.* 5500 kDa in a densitometric scan). On PAGE analysis, no HA less than *ca.* 300 kDa was detected. Vehicle-treated chondrocytes had lower levels of HA, but similarly high molecular mass distribution. This result differs from previous reports of degraded HA being found in conditioned medium from chondrocytes or cartilage explants cultured in the presence or absence of IL-1β [22, 50].

### Enzymatic Degradation of HA by Recombinant Hyaluronidase Was Not Pro-inflammatory

Recombinant rbPH-20 hyaluronidase completely digested HA in chondrocyte cultures within 24 h (Fig. 3b). Conditioned medium was treated with proteinase K, then analyzed by PAGE. HA disappeared from the PAGE gels after digestion, because the resulting HA fragments are smaller than *ca.* 4 kDa, the minimum size for detection by staining HA in the gel.

**Fig. 4:**
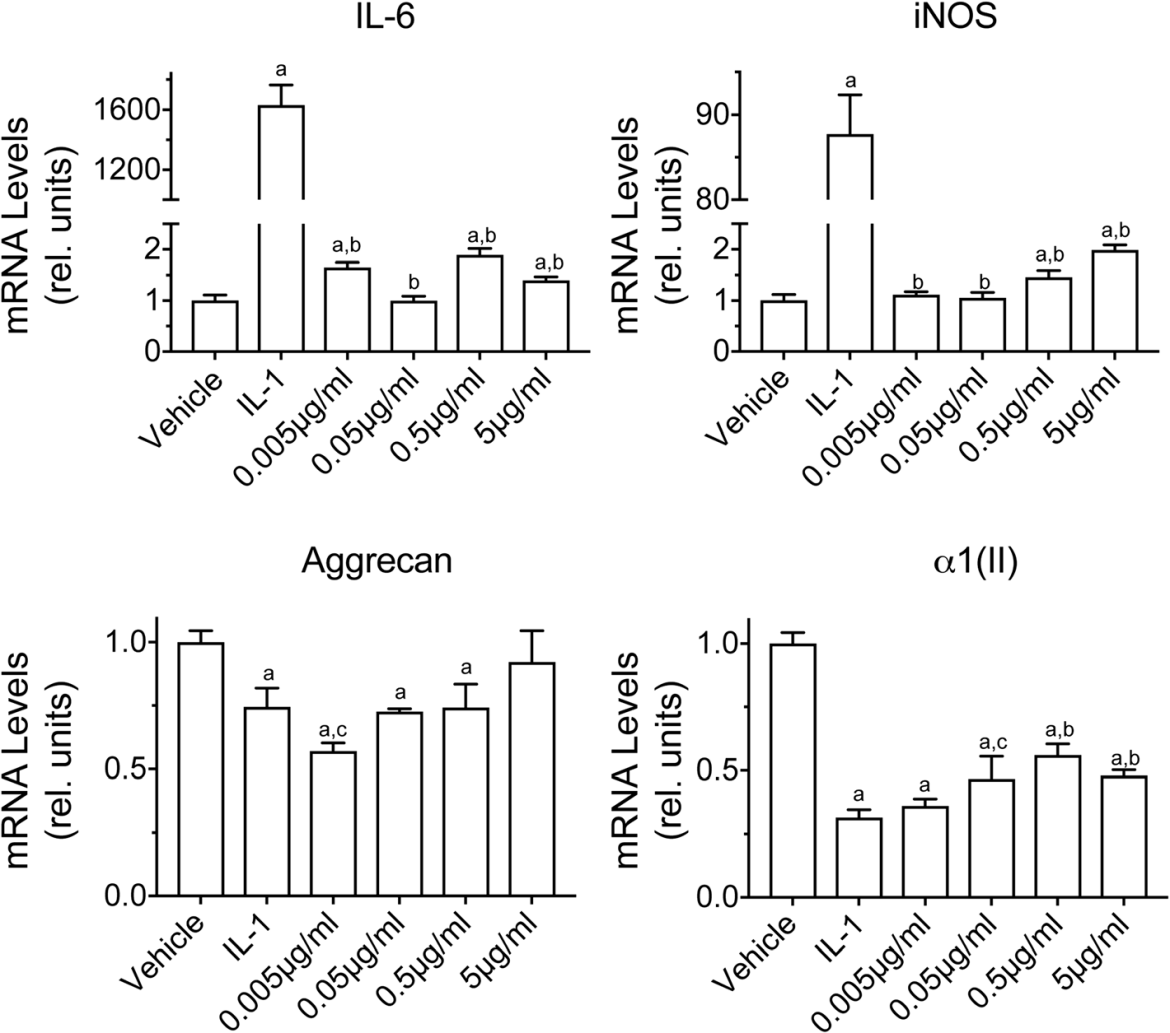
The effect of HA degradation by recombinant bovine PH-20 hyaluronidase at the indicated concentrations on the mRNA levels of catabolic markers (IL-6, iNOS) and articular cartilage markers (aggrecan, type II collagen (α1(II)) in human articular chondrocytes. Human articular chondrocytes were serum-starved for 24 h followed by vehicle treatment (vehicle), treatment with IL-1β (IL-1), or various concentrations of recombinant bovine PH-20 hyaluronidase for 24 h. mRNA levels were determined by real-time PCR using SYBR Green and normalized to 18S RNA. Data were obtained from triplicated PCR reactions using RNA from three different cultures. Values are the mean ± SD. ^a^*p* < 0.01 *vs.* vehicle-treated cells; ^b^*p* < 0.01 *vs.* IL-1-treated cells; ^c^*p* < 0.05 *vs.* IL-1-treated cells.

Degradation of HA to oligosaccharides by rbPH-20 hyaluronidase reduced the mRNA level of type II collagen to a level similar to that caused by IL-1β treatment, while the effect on mRNA levels for aggrecan was small and variable. There was a negligible effect on the mRNA levels of IL-6 or iNOS in comparison with IL-1β treatment (Fig. 4). IL-1β was not expressed in response to rbPH-20 (data not shown). These data suggest that small HA oligosaccharides generated by enzymatic digestion did not have major pro-inflammatory or catabolic effects, when present at the level at which HA is found in human articular chondrocyte cultures.

In contrast to the result for a pure recombinant bovine PH-20, a commercial preparation of isolated bovine testicular hyaluronidase (BTH, PH-20), which completely digested HA in a chondrocyte culture in 24 h, had marked dose-dependent pro-inflammatory and catabolic effects (Supplementary Figs. 2 and 3), suggesting the presence of contaminants in the BTH.

### Exogenous Purified HA Fragments Ranging in Size from Tetrasaccharides to 40 kDa Did Not Have a Pro-inflammatory Effect on Human Articular Chondrocytes

**Fig. 5:**
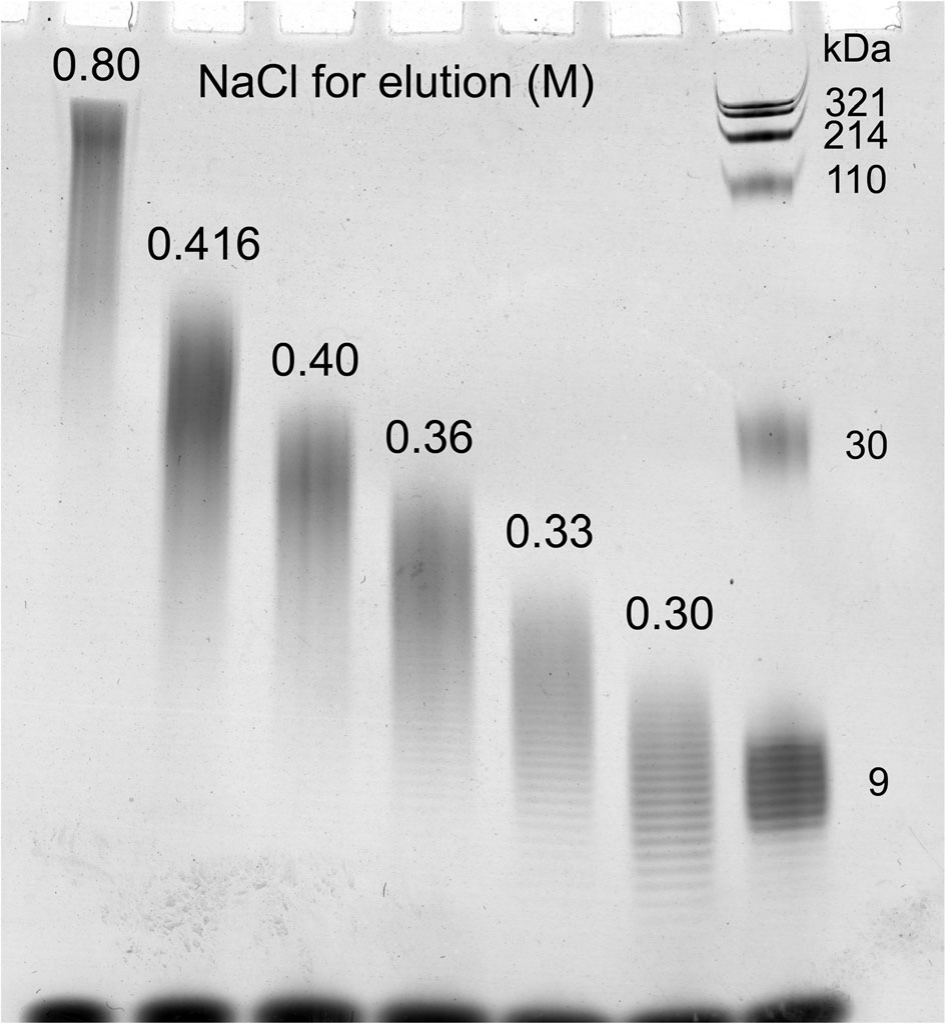
Molecular mass (M) analysis of HA fragments prepared by ion exchange fractionation of a polydisperse mixture of pure low molecular mass (M) HA samples. The M distributions of HA fractions eluted from the column at increasing NaCl concentrations were determined by electrophoresis on 4–20% gradient PAGE and densitometric analysis of the stained gel.

**Fig. 6:**
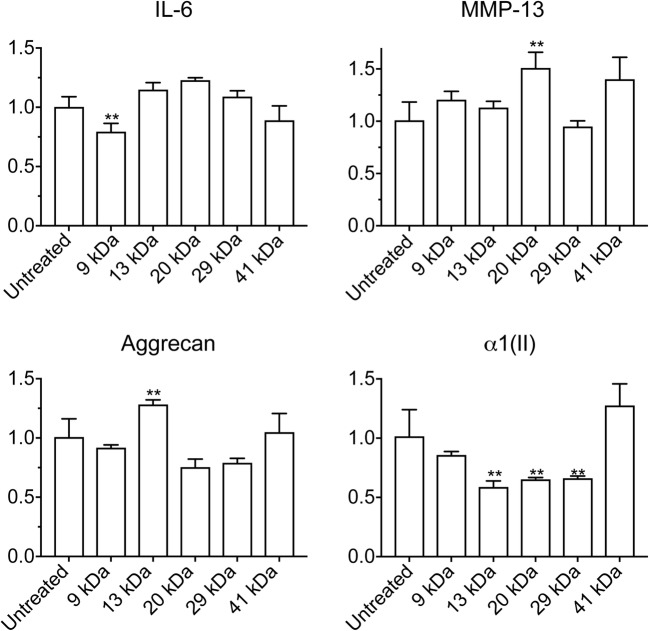
The effect of pure, low-endotoxin HA fragments, ranging in size from 9 to 41 kDa, added to human articular chondrocyte cultures for 48 h at a concentration of 40 μg/mL on the mRNA levels of catabolic markers (IL-6, MMP-13) and articular cartilage markers (aggrecan, type II collagen (α1(II)). Human articular chondrocytes were serum-starved for 24 h followed by treatment with various pure endotoxin-free HA fragments at a concentration of 40 μg/mL for 24 h. mRNA levels were determined by real-time PCR using SYBR Green and normalized to 18S RNA. The mRNA levels are expressed relative to the level of untreated cells, which was set as 1. Data were obtained from triplicated PCR reactions using RNA from three different cultures. Values are the mean ± SD. **p < 0.05 *vs.* untreated cells.

Pure HA fragments with low endotoxin content and low polydispersity were prepared from a highly polydisperse mixture of low M HA samples, using ion exchange (IEX) fractionation. HA M distributions were determined by PAGE (Fig. 5) and densitometric analysis (Supplementary Fig. 4). Five HA fractions, with average M of 9, 13, 20, 29, and 41 kDa, were prepared and found to have low endotoxin levels (Table 1). The HA fractions were added to chondrocyte cultures for 48 h at a final concentration of 40 μg/mL (much higher than the endogenous levels of HA). The low endotoxin HA fragments had no consistent marked effect on the expression of inflammatory and catabolic markers IL-6 and MMP-13 (the effect of 20 kDa HA on MMP-13 expression being an unexplained exception), and the mRNA levels of aggrecan. The 13 kDa, 20 kDa, and 29 kDa HA fragments, however, reduced the mRNA levels of type II collagen (Fig. 6). The high concentration of exogenous HA used in our study may displace high M HA from the cell surface, and the effect on gene expression appears similar to that achieved by enzymatic removal of the endogenous HA.

**Table 1:** Molecular Mass and Endotoxin Content of HA Fractionated by Ion Exchange

NaCl (M) to elute	Average M (kDa)	M range (kDa)^1^	Endotoxin (EU/mg)
0.30	9	7–11	< 0.70
0.33	13	10–16	< 0.14
0.36	20	15–25	< 0.12
0.40	29	23–36	< 0.13
0.416	41	29–54	< 0.05
0.80	> 100	*ca.* 70–200	*ca.* 0.1

^1^Peak width at half height

**Fig. 7:**
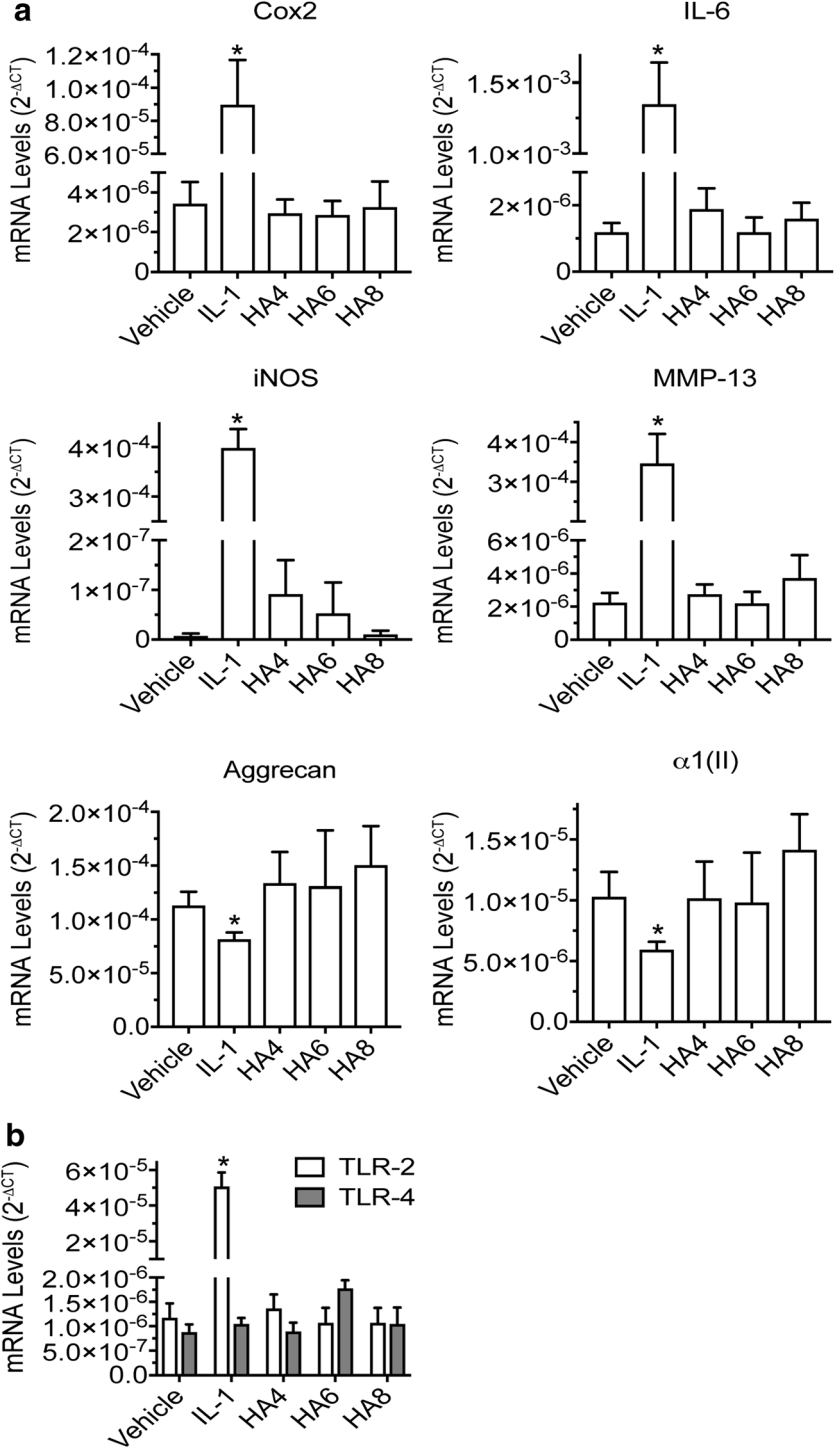
The effect of HA oligos in comparison to IL-1β treatment on the mRNA levels of **a** catabolic markers (Cox-2. IL-6, iNOS, MMP-13) and articular cartilage markers (aggrecan, type II collagen (α1(II)) and **b** TLR2 and TLR4 in human articular chondrocytes. Human articular chondrocytes were serum-starved for 24 h followed by treatment with IL-1β (IL-1), or 4-mer, 6-mer, or 8-mer HA oligos (HA4, HA6, HA8; 40 μg/mL) for 24 h. Control cells were treated with PBS/0.1%BSA vehicle. mRNA levels were determined by real time PCR using SYBR Green and normalized to 18S RNA. Data were obtained from triplicated PCR reactions using RNA from three different cultures. Values are the mean ± SD. *p < 0.01 *vs.* vehicle-treated cells.

Small HA oligosaccharides containing 4, 6, or 8 monosaccharides, all having low endotoxin content, had no significant effect on mRNA levels of catabolic and inflammatory markers (Cox-2, IL-6, iNOS, MMP-13) and also did not decrease expression of cartilage markers (aggrecan, type II collagen) (Fig. 7a). The mRNA levels of TLR2 and TLR4 in our human articular chondrocyte cultures were low and not altered by addition of HA oligosaccharides (Fig. 7b). It is possible that the hexasaccharide and octasaccharide did not effectively displace pericellular HA at the concentration used here (40 μg/mL), which is lower than that used in previous reports [23, 24, 27].

## DISCUSSION

Osteoarthritis is increasingly recognized as a disease in which inflammation plays a role. The presence of IL-1β, in addition to other inflammatory cytokines, contributes to changes in chondrocyte biology and cartilage degradation. Our study examined the effects of IL-1β on HA content and size in chondrocyte cultures, as well as the mRNA levels for proteins involved in HA metabolism and interactions that are important to maintenance of the chondrocyte pericellular matrix. In addition, the potential role of endogenous or exogenous HA fragments in exacerbating inflammation was examined.

The mRNA levels of genes for HA synthases HAS2 and HAS3 were increased by treatment of human articular chondrocytes with IL-1β, but there was little or no change in the mRNA levels of the hyaluronidase Hyal2. mRNA for other hyaluronidases were present at low or undetectable levels. As expected on the basis of these results, HA-specific assay of conditioned medium showed an increase in HA content due to IL-1β treatment. The increased synthesis of HA was not accompanied by a reduction in the average size of HA. We observed HA present in the conditioned medium of human articular chondrocytes stimulated by IL-1β to have a high average M of approximately 5.5 MDa, and we did not detect any HA below 300 kDa. This suggests that the increased rate of HA synthesis was sufficient to combat any increase in degradative agents and processes. It is important to note that chondrocytes cultured alone may not produce hydroxyl radicals in the absence of exogenous ferrous iron, or superoxide anion needed to form peroxynitrite, whereas *in vivo* other sources of these agents (*e.g.*, macrophages, hemoglobin iron) could contribute, and lead to HA degradation .

Previous studies have reported that HA fragments can act as alarmins and cause inflammatory and catabolic responses in various cell types, including articular chondrocytes [7, 10–17, 23, 24, 27, 28]. Other studies, however, reported no inflammatory and catabolic responses caused by HA fragments in a variety of cells, including human monocytes, synovial fibroblasts, and chondrocytes [18, 19]. Concerns about possible endotoxin contamination in the HA preparations used in studies in which an inflammatory effect of HA fragments on cells was observed have been raised [18]. In our study, degradation of endogenous HA by a pure recombinant hyaluronidase, or addition of pure low-endotoxin HA oligosaccharides (4-,6-, 8-mers) or larger HA fragments averaging 9 to 41 kDa to cultured human articular chondrocytes did not strongly or consistently induce the expression of catabolic and inflammatory markers. A possible explanation for the variability in the effect of HA fragments on cells is that the cellular response is dependent on cell type and the status of the cellular metabolism and microenvironment. In this respect, it should be noted that the human articular chondrocytes used in this study had low mRNA levels of RHAMM and TLR4, two proteins associated with defensive or pro-inflammatory signaling in the presence of HA fragments [14, 25, 26, 51, 52]. More importantly, the low mRNA levels of RHAMM and TLR4 were not significantly altered or were further decreased in IL-1β-treated chondrocytes.

There are important changes in expression of HA-binding proteins of the pericellular matrix in chondrocyte cultures under inflammatory stimulus, which may affect cellular response. Aggrecan expression was observed to decrease in chondrocytes exposed to IL-1β, in agreement with previous reports [38, 40, 43, 53]. The normal homeostatic ratio of HA to aggrecan is therefore dysregulated, as HA levels are increased and aggrecan levels decreased. Inflammation would thus cause pathological weakening of the integrity of the pericellular matrix. Other HA-binding proteins could provide compensatory interactions to stabilize the cellular coat under inflammation. Cultured human articular chondrocytes exposed to IL-1β showed increased expression of CD44 and TSG-6. The increased CD44 expression may reflect a connection with the increase in HA synthesis, and serve to hold the higher amount of HA present. TSG-6, an anti-inflammatory response protein, has the most strongly increased expression. TSG-6 is known to noncovalently bind and crosslink HA by TSG-6 self-association (enhancing HA binding to CD44), as well as to catalyze the covalent transfer of HC domains from IαI to HA, further crosslinking HA [54]. These observations suggest that the increased expression of TSG-6 observed here could result in stabilization of the pericellular HA matrix, providing an enhanced protective shield for cells.

In conclusion, this study showed that HA fragments were not produced by IL-1β-stimulated human articular chondrocytes in the absence of other sources of reactive oxygen or nitrogen species, and that exogenous HA fragments from oligosaccharides up to about 40 kDa in molecular mass were not significant pro-inflammatory agents for human articular chondrocytes, probably due to low expression of TLR4 and RHAMM in these cells. The chondrocyte response to IL-1β included an increased synthesis of high molecular mass HA, even as the expression of aggrecan was reduced. Compensatory increases in expression of CD44 and TSG-6 suggest that the defensive response of the chondrocytes may involve alternative HA crosslinking mechanisms to support the pericellular matrix integrity. These observations suggest new directions for investigation, regarding the role of HA and HA-binding proteins in maintenance of the chondrocyte pericellular matrix, and in their contribution to disease progression or resolution in the joint.

## Electronic supplementary material

ESM 1 (PDF 272 kb)

## Abbreviations

BSA: Bovine serum albumin
BTH: Bovine testicular hyaluronidase
Cox: Cyclooxygenase
DMEM: Dulbecco’s modified Eagle’s medium
ECM: Extracellular matrix
EDTA: Ethylenediaminetetraacetic acid
ELISA: Enzyme-linked immunosorbent assay
EU: Endotoxin units
EV: Extracellular vesicles
FCS: Fetal calf serum
GAG: Glycosaminoglycan
HA: Hyaluronan
HAS: Hyaluronan synthase
HC: Heavy chains of inter-α-inhibitor
HYAL: Hyaluronidase
IαI: Inter-α-inhibitor
IEX: Ion exchange
IL: Interleukin
iNOS: Inducible nitric oxide synthase
LAL: Limulus amebocyte lysate
LPS: Lipopolysaccharide
M: Molecular mass
MMP: Matrix metalloproteinase
NTA: Nanoparticle tracking analysis
OA: Osteoarthritis
PAGE: Polyacrylamide gel electrophoresis
PBS: Phosphate-buffered saline
rbPH-20: Recombinant bovine PH-20 hyaluronidase
RHAMM: Receptor for hyaluronan-mediated mobility
TBE: Tris borate EDTA buffer
TLR: Toll-like receptor
TNF-α: Tumor necrosis factor-alpha
TSG-6: Tumor necrosis factor-inducible gene 6 protein
