# Supplementary material for: Role of Hyaluronan in Inflammatory Effects on Human Articular Chondrocytes
Source: Inflammation. 2019 Jun 27;42(5):1808–20. doi: 10.1007/s10753-019-01043-9 (PMC6719336; doi:10.1007/s10753-019-01043-9)
Supplement: Supplementary file 1 — (PDF 272 kb) [file 10753_2019_1043_MOESM1_ESM.pdf]

Cowman et al.

Role of Hyaluronan in Inflammatory Effects on Human Articular Chondrocytes

Supplementary Figures

Supplementary Figure 1. EVs released into the medium from human articular chondrocytes in the absence or presence of IL-1β. Human articular chondrocytes were serum starved for 24h followed by treatment with IL-1β (10ng/ml) in PBS/0.1%BSA (IL-1) or PBS/0.1%PBS (Vehicle) for 48h. The numbers of EVs released into the medium of IL-1β-treated or vehicle-treated cells were analyzed by nanoparticle tracking analysis and normalized to the total cell number. Data were obtained from four different experiments and expressed as mean ± SD. *$p < 0.01$ vs. vehicle-treated cells.

Supplementary Figure 2. Treatment of cultured human articular chondrocytes with commercial isolated bovine testicular hyaluronidase (BTH) at 10 U/ml for 24 h degraded all HA in the conditioned medium, as shown by 4-20% gradient PAGE.

Supplementary Figure 3. The effect of HA degradation by a commercial preparation of isolated bovine testicular hyaluronidase (BTH, PH-20), at the indicated concentrations on the mRNA levels of catabolic markers (IL-6, iNOS) and articular cartilage markers (aggrecan, type II collagen (α1(II)) in human articular chondrocytes. Human articular chondrocytes were serum-starved for 24h followed by treatment with various concentrations of a commercial preparation of isolated bovine testicular hyaluronidase (BTH, PH-20) for 24h. mRNA levels were determined by real time PCR using SYBR Green and normalized to 18S RNA. Data were

obtained from triplicated PCR reactions using RNA from three different cultures. Values are the mean $\pm$ SD. *p < 0.01 vs. untreated cells; **p < 0.05 vs. untreated cells.

Supplementary Figure 4. Densitometric Analysis of molecular mass (M) distributions for HA fragments prepared by ion exchange fractionation of a polydisperse mixture of pure low molecular mass (M) HA samples. The M distributions of HA fractions eluted from the column at increasing NaCl concentrations were determined by electrophoresis on 4-20% gradient PAGE followed by densitometric analysis of the stained gel.

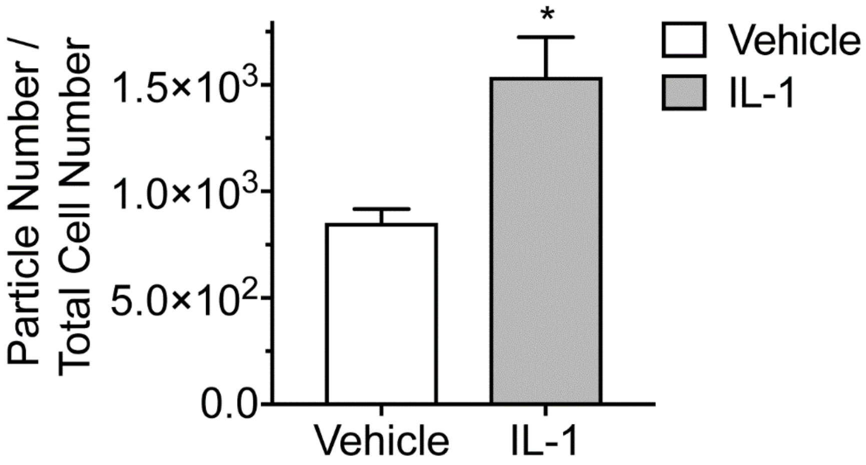

Supplementary Figure 1

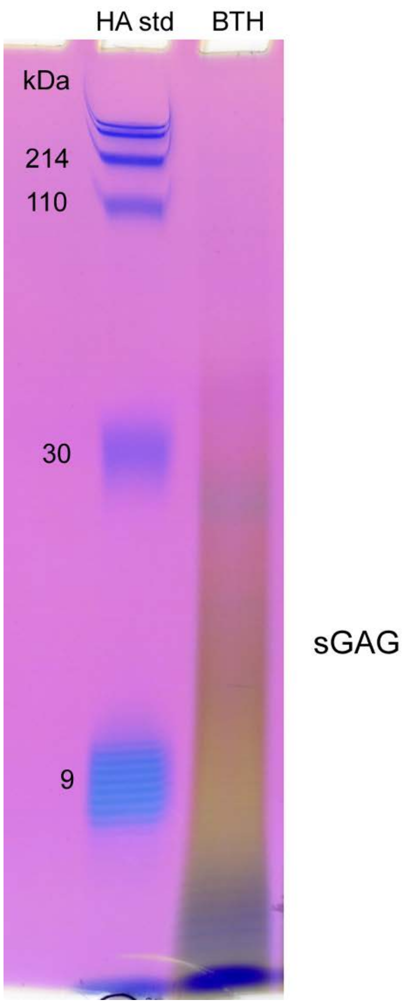

Supplementary Figure 2

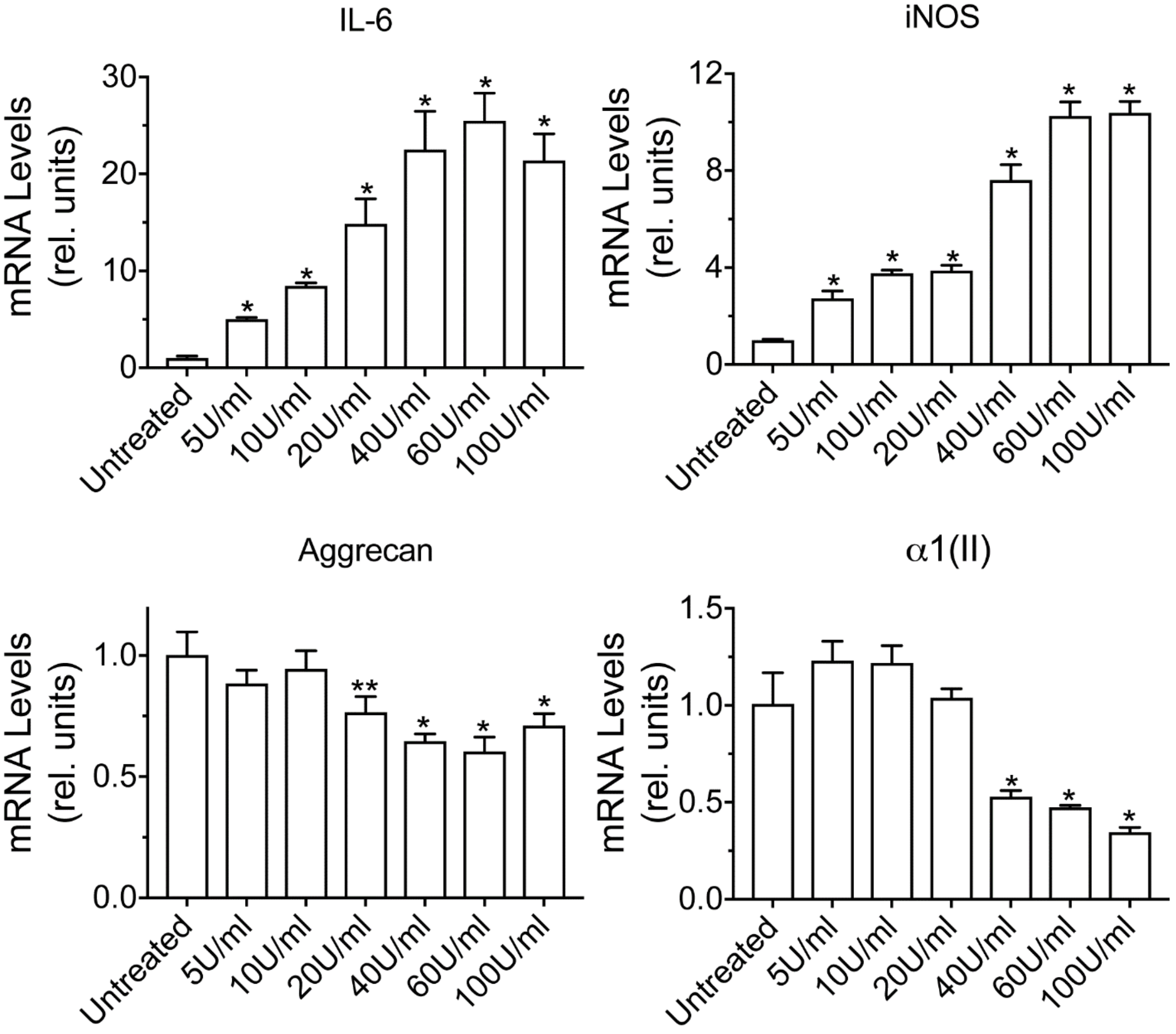

Supplementary Figure 3

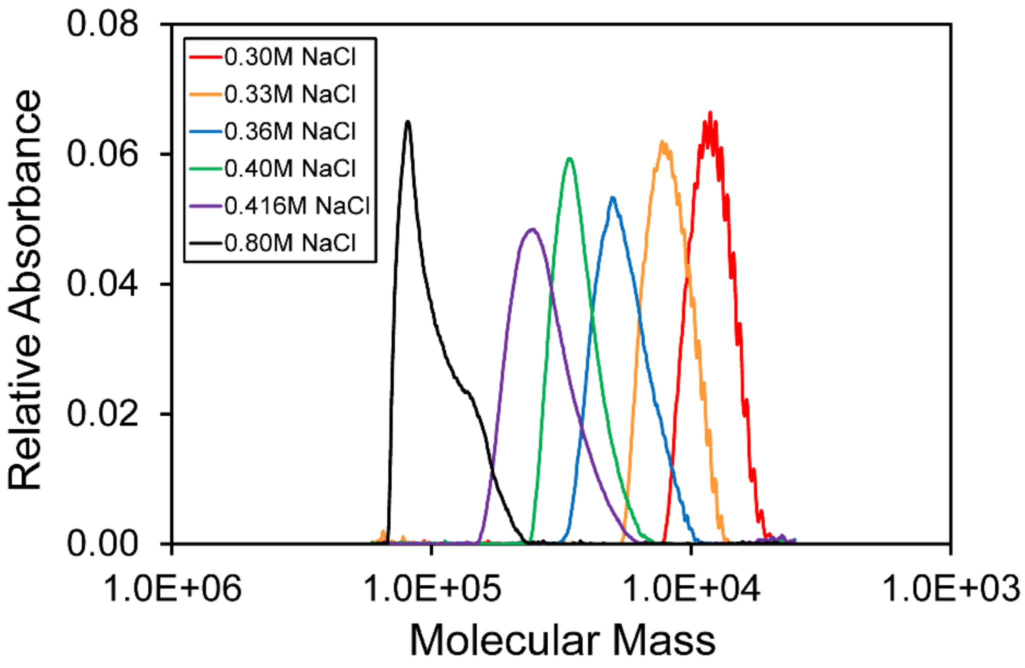

Supplementary Figure 4
